# On The Potential of Image Moments for Medical Diagnosis

**DOI:** 10.3390/jimaging9030070

**Published:** 2023-03-17

**Authors:** Cecilia Di Ruberto, Andrea Loddo, Lorenzo Putzu

**Affiliations:** 1 Department of Mathematics and Computer Science, University of Cagliari, Via Ospedale 72, 09124 Cagliari, Italy; 2 Department of Electrical and Electronic Engineering, University of Cagliari, Piazza d’Armi, 09123 Cagliari, Italy

**Keywords:** medical diagnosis, medical imaging, image classification, feature extraction, moments

## Abstract

Medical imaging is widely used for diagnosis and postoperative or post-therapy monitoring. The ever-increasing number of images produced has encouraged the introduction of automated methods to assist doctors or pathologists. In recent years, especially after the advent of convolutional neural networks, many researchers have focused on this approach, considering it to be the only method for diagnosis since it can perform a direct classification of images. However, many diagnostic systems still rely on handcrafted features to improve interpretability and limit resource consumption. In this work, we focused our efforts on orthogonal moments, first by providing an overview and taxonomy of their macrocategories and then by analysing their classification performance on very different medical tasks represented by four public benchmark data sets. The results confirmed that convolutional neural networks achieved excellent performance on all tasks. Despite being composed of much fewer features than those extracted by the networks, orthogonal moments proved to be competitive with them, showing comparable and, in some cases, better performance. In addition, Cartesian and harmonic categories provided a very low standard deviation, proving their robustness in medical diagnostic tasks. We strongly believe that the integration of the studied orthogonal moments can lead to more robust and reliable diagnostic systems, considering the performance obtained and the low variation of the results. Finally, since they have been shown to be effective on both magnetic resonance and computed tomography images, they can be easily extended to other imaging techniques.

## 1. Introduction

Medical imaging is widely used to manage various types of pathologies for diagnosis and postoperative or post-therapy monitoring. As a result, the number of biomedical images has increased significantly, meaning that pathologists and doctors spend much time searching for diseases in images that could be used to treat patients. This is even more important considering that about 80% of biomedical images turn out to be benign [1]. Moreover, the manual interpretation of biomedical images is a time-consuming and challenging task that requires experienced operators, but the results are still subjective, influenced by the operator’s competence or level of fatigue. In contrast, accuracy and efficiency are crucial in this field. Therefore, the development of automated tools capable of effectively handling this massive number of images has become essential.

Computer-aided diagnosis (CAD) systems can assist pathologists and physicians in diagnosing and postoperative or post-therapy monitoring. Such systems attempt to mimic manual inspection [2] but can provide accurate results while reducing subjectivity and speeding up the analysis process [3]. The growing need for automated approaches has attracted the attention of many researchers in this field, who have proposed several CAD systems based on image processing and machine learning techniques [4,5].

Existing CAD systems can be more or less complex, depending on the medical task and the type and quality of images to be analysed. In this study, we focused on methods for direct image classification, which consists of two main steps: feature extraction and classification. The first step generally consists of extracting a set of parameters from an image to characterise the shapes, colour, and texture it contains [6]. In the second step, the extracted features are used to build a model of known cases (during the training phase) and provide a diagnosis of unknown cases (during the testing phase).

Since the advent of convolutional neural networks (CNNs) and in particular, with the proposal of the most famous AlexNet [7] and very deep convolutional networks (VGGNet) [8], many authors have focused on this approach as the sole method of diagnosis. In fact, CNNs can perform feature extraction and classification steps simultaneously because of their internal structure.

Specifically, CNNs can be used as feature extractors by extracting activations from a single layer or multiple layers that can globally describe the images [9]. Such features can then be used to train standard machine learning models, such as ensemble techniques [10]. The use of CNNs in CAD systems dramatically simplifies the design and analysis work but also leads to the development of much more complex CAD systems whose results are often challenging to interpret [11]. For these reasons, many CAD systems still rely on handcrafted features, especially global image descriptors such as texture features and orthogonal moments [6,9,10]. Texture feature methods compute simple or more sophisticated statistical properties, e.g., co-occurrence matrices [12,13,14] or local binary patterns (LBP) [15]. Orthogonal moments are statistical measures for describing objects in images regardless of their position, viewing angle, and illumination, due to their ability to represent global features of an image [16]. In addition, they are less sensitive to noise and can represent the properties of an image without redundancy [17,18].

Due to their significant properties, orthogonal moments have been widely used in various tasks ranging from edge detection [19] to image classification [20]. In particular, they have been applied in several CAD systems, from the diagnosis of breast masses in mammographic images [21] to the analysis of liver textures in computed tomography (CT) scans [22] or the study of ultrasound images of the prostate [23].

The first moments to be introduced were Hu’s moments [24] in 1962, and since then, interest in this type of descriptor has grown considerably. Many works have followed to propose different kinds of moments (circular or Cartesian) [16] or to present more efficient and, at the same time, more accurate formulations [25].

In this work, we perform a detailed comparison between all the best-known types of orthogonal moments in order to evaluate their effectiveness in diagnostics when applied to very different medical images. This work is the first in which the performance of different moments is compared on the same tasks. Indeed, most works in the literature exploit just single moments [21,26,27,28,29,30] or a combination of moments [23,31,32,33] to solve a particular problem. For completeness, we compare the performance of the moments with the best-known texture features and deep features (extracted from pretrained CNNs). It is important to note that deep features are used in this work without any explicit training or fine-tuning of new learners.

Our contribution is fourfold: *(i)* we reviewed the state of the art on orthogonal moments by describing the main features and differences between them; *(ii)* we summarised the main types of moments, emphasising their differences; *(iii)* we examined and compared orthogonal moments in several medical diagnostic tasks; and *(iv)* we compared orthogonal moments with the best-known texture features and the latest CNNs features. Our numerical experiments allow us to identify the most effective category of moments for the tasks under consideration and also show a particularly encouraging behaviour. Indeed, they confirm that CNNs perform excellently in all tasks, but orthogonal moments seem competitive with them. The orthogonal moments have very little resource consumption, so they can be considered a great alternative to well-known CNN-based descriptors. The rest of this paper is organised as follows. Section 2 discusses related work and the role of orthogonal moments in the medical field. In Section 3, we describe orthogonal moments, their categorisations, and their main properties. In Section 4, we describe the materials and methods used in the experimental evaluation, then we present the results in Section 5.2 and discuss them in Section 5.3. Finally, in Section 6, we summarise the content of the article and outline directions for future work.

## 2. Related Works

The moments are statistical measures used to characterise a function, capture its most important features, and thus obtain relevant information about an object. Hu was the first to use the invariants of geometric moments in pattern recognition [24] and demonstrated the discriminative power of these features in the case of recognition of printed capital letters. Non-orthogonal moments have lost their attractiveness in favour of orthogonal moments constructed in terms of orthogonal polynomials because the non-orthogonal property of these invariant geometric moments causes information redundancy. It has been shown that orthogonal moments are less sensitive to noise and have an efficient ability to represent features with a low degree of information redundancy. Among the best-known are the discrete Chebyshev moments [34], the discrete Krawtchouk moments [35], the Legendre moments [36], and the Zernike moments [37]. For these reasons, orthogonal moments have received a great deal of attention in recent years, and a large number of novel moments have been proposed.

Recent surveys, such as those conducted by Qi et al. [38] and Singh et al. [16], have reviewed various families of orthogonal moments, their theoretical foundations, implementation methods, and evaluation techniques for measuring their performance in various image analysis tasks. In particular, the latter conducted a comprehensive review of methods for achieving rotation invariance in orthogonal moments and transforms used for image representation, including using rotationally invariant functions, the introduction of rotation parameters, and the use of multiple basis functions.

Image moments have proven to be versatile tools with numerous applications. In quality assessment tasks, moments have been successfully used for the evaluation of authentically distorted images [39]. Additionally, they have also been applied to the recognition of symmetric objects [40], which can be beneficial in robotics and object recognition applications in computer vision, image compression [41], or image watermarking [42].

However, moments find their most extensive application in the medical field. Their usage encompasses a wide range of tasks, including the reconstruction of medical images afflicted by noise, such as CT, magnetic resonance (MR), and X-ray images [43], the detection of pathological conditions, such as brain tumours [44], and the classification of medical images. Classification examples include the work of Wu et al. [23], which used moments to describe the texture of CT liver images and to detect tumours in ultrasound prostate images. Similarly, Iscan et al. [45] and Tahmasbi [21] used moments to detect tumours in brain and mammography images, respectively. Further examples include the works using moments to classify parasites in microscopy images by Dogantekin et al. [46] and spermatogonium by Liyun et al. [47].

In recent decades, the use of moments as features in various applications has become widespread. This popularity can be attributed to their ability to represent an image’s salient features effectively. Numerous studies have employed different categories of moments to serve as image descriptors to accomplish specific tasks. For example, Hu, Legendre, and Zernike moments were investigated and compared to automatically classify boar spermatozoa acrosomes as intact or damaged [48]. In that work, images of boar spermatozoa were then classified by k-nearest neighbour (k-NN) and multilayer perceptron classifiers. Instead, in classifying histological images, Di Ruberto et al. [49] realised a comparative evaluation of different moments, such as Hu, Legendre, and Zernike’s, and texture features, such as local binary patterns and Haralick features. The best results are reported when the orthogonal moments are combined with co-occurrence matrices.

As far as Zernike moments are concerned, on the one hand, Tahmasbi et al. [21] used them as descriptors for identifying breast masses based on their shape and edge characteristics. The study involved extracting two sets of Zernike moments from preprocessed images, each consisting of 32 moments with varying orders and iterations, and using them at the feature selection stage. On the other hand, pseudo-Zernike moments were used to classify leukocyte subtypes in Ryabchykov et al. [26], whereas Elaziz et al. [27] applied fractional-order orthogonal descriptors for bacterial species recognition.

Regarding Chebyshev moments, they were evaluated by Mukundan [28]. The authors explored 5×5 neighbourhoods of pixels to encode texture information as a Lehmer code, representing the relative strengths of the moments considered. The generated local descriptors were then used to classify tissue images into six classes.

Krawtchouk moments were used alone for blind integrity verification of medical images in Xu et al. [29] and combined in the extensive work of Batioua et al. [30]. The authors propose new sets of separable discrete moments for 3D image analysis called TKKM (Tchebichef–Krawtchouk–Krawtchouk moments) and TTKM (Tchebichef–Tchebichef–Krawtchouk moments). The study indicated that the proposed 3D separable moments significantly improved representation capabilities compared to traditional Krawtchouk and Tchebichef moments.

Although Hu moments have the well-known problems of noise sensitivity and information redundancy caused by the nonorthogonality of the geometric basis [30], they have been used with equal success. To cite some examples, Siti et al. [50] employed them to classify breast cancer, Laine et al.applied Hu moments to classify the structure of large populations of biopharmaceutical viruses at high resolution [51], and, more recently, in [52], Hu moments were extracted from segmented ultrasound arterial wall images for assessing the risk of atherosclerosis and predicting the chance of myocardial infarction.

To overcome the limitations of Hu moments and improve the performance further, several works proposed combining heterogeneous types of moments, even with different categories of descriptors. For example, in [31], Hu moments were combined with VGG16, an off-the-shelf CNN, to automatically screen COVID-19 based on CT scans. Concerning other combinations, Wu et al. [23] combined Legendre, Zernike, Krawtchouk, and Chebyshev moments to classify CT liver images. In contrast, Lao et al. [32] proposed a framework for Alzheimer’s diagnosis based on combining the 3D discrete wavelet transform and 3D moment invariants features from multimodality images. These features were first provided to a deep neural network with stacked autoencoders for dimensionality reduction, and finally, a softmax regression made the final classification.

Furthermore, the combination of moments appeared beneficial also to improve and speed up the medical image reconstruction of MR, CT, and mammography, as shown in [33]. In that work, an effective method for analysing different types of 2D and 3D medical images based on the discrete orthogonal moments of Chebyshev, Krawtchouk, Hahn, Charlier, and Meixner was presented.

Image moments have generally demonstrated versatility and usefulness in various domains and applications. This makes them a valuable tool in the field of computer vision and image processing, particularly with regard to medical tasks.

## 3. Overview of Moments

Moments can be defined as the projections of an image function onto particular kernel functions and, mathematically, can be defined as follows.

A general moment $M_{pq}^{f}$ of an image f(x,y), where p,q are non-negative integers and (p+q) is called the *order* of the moment, is defined as in Equation (1) [53]:

(1) $$M_{pq}^{f}=\int \int_{D}h_{pq}\left(x,y\right)f\left(x,y\right)dxdy$$

where $h_{pq}\left(x,y\right)$ is the kernel function (also known as the polynomial basis function) defined on *D*.

**Figure 1:**
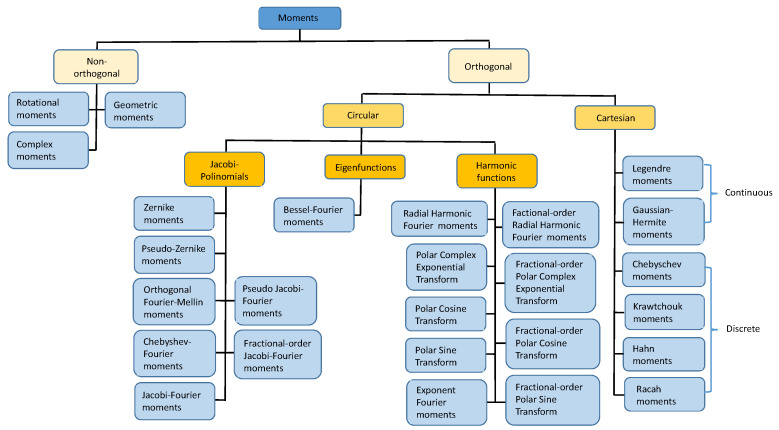
A schematic classification of image moments.

According to the mathematical properties of the polynomial basis used, we can recognise various categories of moments, as shown in Figure 1.

Depending on whether the basis functions satisfy orthogonality, the image moments can be classified into **orthogonal moments** or **non-orthogonal moments**. Orthogonality means that two different basis functions of the set of basis functions are unrelated or are “perpendicular” in geometric terms, which leads to no redundancy in the set of moments. Some of the most popular **non-orthogonal moments** are *geometric moments* [54], *rotational moments* [55], and *complex moments* [56].

Due to the nonorthogonality of the basis functions, a high information redundancy and high noise sensitivity exist in such moments. As a result, image reconstruction from these moments is quite difficult, and the image representation power is poor. In contrast, orthogonal moments, built on a set of orthogonal polynomials, have received much attention in recent years due to their ability to represent images with minimal information redundancy and a high noise robustness [25]. Orthogonal moments can be divided into those defined in either **Cartesian** coordinates, (x,y), or **polar** coordinates, (r,θ).

Orthogonal moments defined in polar coordinates are also called radial or circular orthogonal moments. The kernel function of radial orthogonal moments is built on a particular type of radial orthogonal polynomial and a complex exponential angular Fourier factor. In addition to their image reconstruction property, the significant advantage of radial orthogonal moments lies in their ability to achieve rotation invariance. In this regard, there are mainly three types of orthogonal functions used as definitions, including *Jacobi polynomials*, *harmonic functions*, and *eigenfunctions*.

**Circular orthogonal moments based on Jacobi polynomials** mainly include *Zernike moments* [37], *pseudo-Zernike moments* [55], *orthogonal Fourier–Mellin moments* [57], *Chebyshev–Fourier moments* [58], *Jacobi–Fourier moments* [59], and *pseudo Jacobi–Fourier moments* [60].

Among the **circular orthogonal moments derived from harmonic functions**, there are the *radial harmonic Fourier moments* [61], *exponent Fourier moments* [62], polar harmonic-transform-based moments [63], i.e., *polar complex exponential transform*, *polar cosine transform*, and *polar sine transform*. The most representative moments based on **eigenfunctions** are the *Bessel–Fourier moments* [64].

The kernel functions of orthogonal moments defined in Cartesian coordinates are constructed on normal orthogonal polynomials. The orthogonal moments defined in Cartesian coordinates can be further divided into two types, **continuous** and **discrete** orthogonal moments, depending on whether the kernel function $h_{pq}\left(x,y\right)$ is orthogonal on the continuous or discrete domain. The **continuous** orthogonal moments mainly include *Legendre moments* [37] and *Gaussian–Hermite moments* [65], while the **discrete** orthogonal moments are mainly composed of *Chebyshev moments* [34], *Krawtchouk moments* [35], *Hahn moments* [66], and *Racah moments* [67].

All of the orthogonal moments mentioned above are restricted to being of integer order since orthogonal polynomials should be of integer order $\left(p,q\right)\in Z^{2}$. In recent years, there has been a focus on investigating fractional-order domain [68,69,70]. In such domain, a fractional-order parameter α∈R is introduced through certain suitable variable substitution, e.g., $x\leftarrow x^{\alpha }$, $y\leftarrow y^{\alpha }\in \left[0,1\right]$ in Cartesian moments, and $r\leftarrow r^{\alpha }\in \left[0,1\right]$ in circular moments. Thus, the order of the newly defined moments can be extended to the real domain, e.g., $\left(\alpha p,\alpha q,\right)\in R^{2}$ for Cartesian moments and αr∈R for circular moments, although so far, mostly the circular moments have been studied and extended to the fractional domain [70,71], even in combination with colour information [72]. The fractional-order category includes radial harmonic Fourier moments, the polar complex exponential transform, the polar cosine transform, the polar sine transform and Jacobi–Fourier moments. They can be described by a recurrence relation which guarantees a high noise robustness in invariant image recognition [70,71].

In Table 1, we summarised the orthogonal moments analysed in this work, with the appropriate reference and the year in which they were first proposed.

## 4. Medical Image Classification

This section describes the materials and methods used to compare the above-mentioned moment features for CT and MR images classification. In the following, we first describe the data sets used in Section 4.1. Then, in Section 4.2, we list the moments and other features used for comparison purposes and finally, the classification models in Section 4.2.

### 4.1. Data Sets

**Figure 2:**
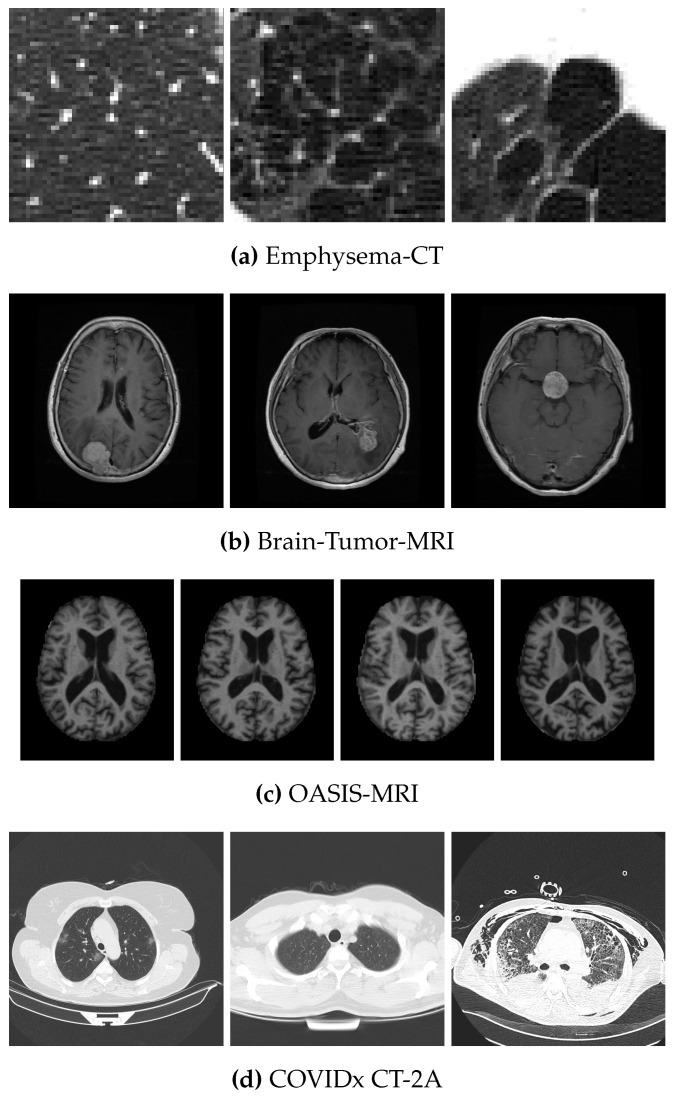
Illustrations of the data sets classes. From left to right, (**a**) shows normal tissue, centrilobular emphysema, and paraseptal emphysema; in (**b**): meningioma, glioma, and pituitary tumours. (**c**) represents four classes of dementia: absent, very mild, mild, and moderate. Finally, (**d**) includes COVID, normal, and pneumonia.

We now describe the four different reference data sets used in this work. Each one is publicly available. One sample per class is illustrated in Figure 2. **Emphysema-CT** [74] is a database of CT images created to develop texture-based CT biomarkers for detecting and diagnosing chronic obstructive pulmonary diseases related to emphysema. It includes 115 high-resolution CT sections from which 168 square patches (of size 61×61) were manually cropped and annotated by experienced clinicians into three main categories: normal tissue, centrilobular emphysema, and paraseptal emphysema, consisting of 59, 50, and 59 samples, respectively.

**Brain-Tumor-MRI** [75] is a data set of MR images containing different brain sections from 233 patients, created to develop CAD systems to diagnose the presence of brain tumours. Here, we used the axial sections subset containing 870 images of size 512×512, featuring three types of brain tumours: meningioma, glioma, and pituitary tumour. They are present in 143, 436 and 291 samples, respectively.

**OASIS-MRI** [76] is also an MR image data set containing different brain sections but was collected to study the clinical dementia rating. Again, we focused only on the axial section images, for a total of 436 images of size 176×208, divided into 336 representing cognitively healthy individuals and 100 with subtypes of dementia (70 with very mild, 28 with mild, and 2 with moderate dementia).

**COVIDx CT-2A** [77] consists of 194,922 CT images of 3745 patients from 15 different countries, aged 0–93 years (median age 51). Specifically, the CT images are provided with size 512×512 pixels and are annotated into three different classes: novel coronavirus pneumonia due to SARS-CoV-2 viral infection, common pneumonia, and normal condition. The available CT images were 3253, 873, and 816, respectively, for a total of 4942 images.

The summarising details for each data set are reported in Table 2.

### 4.2. Methods

We evaluated different kinds of invariant moments in our experiments for a total of 20 descriptors that we grouped into four important classes, following what we reported in Section 3:

- Cartesian moments: continuous Legendre moments (LM), discrete Chebyshev moments of the first order (CHM), discrete Chebyshev moments of the second order (CH2M).
- Circular Jacobi polynomials based moments: Zernike moments (ZM), pseudo-Zernike moments (PZM), orthogonal Fourier–Mellin moments (OFMM), Chebyshev–Fourier moments (CHFM), pseudo-Jacobi–Fourier moments (PJFM), Jacobi–Fourier moments (JFM), fractional-order Jacobi–Fourier moments (FrJFM).
- Circular eigenfunction-based moments: Bessel–Fourier moments (BFM).
- Circular harmonic-function-based moments: radial harmonic Fourier moments (RHFM), exponent Fourier moments (EFM), polar complex exponential transform (PCET), polar cosine transform (PCT), polar sine transform (PST), fractional-order radial harmonic Fourier moments (FrRHFM), fractional-order polar complex exponential transform (FrPCET), fractional-order polar cosine transform (FrPCT), fractional-order polar sine transform (FrPST).

A subset of the orthogonal moments representative of all the classes presented in the taxonomy was chosen for this analysis. The order was set to 5 for all the moments. A higher-order would have added irrelevant features or noise [25]. In addition, as mentioned earlier, we also compared the performance of orthogonal moments with some texture and deep features for completeness.

The texture features computed were the rotation invariant grey-level co-occurrence matrix (GLCM) features as proposed in [78] and rotation-invariant LBP features [79]. GLCMs were computed with d=1 and $\theta =[0^{\circ },45^{\circ },90^{\circ },135^{\circ }]$. We extracted thirteen rotation-invariant features $Har_{ri}$ [78] from them. The LBP map was extracted in the neighbourhood identified by *r* and *n* equal to 1 and 8, respectively, and then converted to a rotationally invariant feature vector $LBP_{ri}$ [79].

The deep features were extracted from four off-the-shelf network architectures: AlexNet [7], VGG19 [8], ResNet50 [80], and GoogLeNet [81]. In both AlexNet and VGG19, we extracted features from the penultimate fully connected layer (fc7), which created a feature vector of size h=4096, while for ResNet50 and GoogLeNet, we extracted features from the only fully connected layer present, which created a feature vector of size h=1000. Since all referenced CNN architectures were pretrained on the vast image data set ImageNet [82], they had already learned sufficient representation and generalisation capability for various visual recognition tasks [83,84]. Therefore, we directly extracted the features without a fine-tuning process on the target data sets [83].

In order to perform a more detailed evaluation, the results of which are not related to the capabilities of the individual classifier, we chose a single classification technique from each of the following categories: numerical function models, instance-based models, symbolic or logical models, probabilistic-based models and ensemble models. Therefore, we selected five different classification models: support vector machine (SVM) [85], k-NN [86], decision trees (DT) [87], naive Bayes (NB) [88] and bagged trees (BT) [88].

## 5. Experimental Results

This section describes the experimental evaluation that was conducted and the results that were obtained. The purpose of this study was to analyse the performance of moment-based features for the classification of medical images, as previously mentioned. For this purpose, we conducted various experiments that involved the mentioned data sets, from which the features were extracted and then used to build and test different classification models.

### 5.1. Experimental Setup

To ensure a fair comparison, the experimental evaluation was performed using a model validation procedure consisting of a k-fold cross-validation applied to the entire data set. The training fold was used for data normalisation and hyperparameter optimisation for each cross-validation. Therefore, the best hyperparameters were optimised for all classifiers using a grid search.

Although we were not interested in the absolute best performance in terms of classification, the optimisation process was repeated for each individual descriptor to determine the best setting for each descriptor and its upper bound in terms of performance.

The classification performance was evaluated using only test fold data; the k-fold cross-validation was repeated with *k* equal to 10. The performance of the learning models was measured in terms of accuracy. The data sets were multiclass, so the accuracy was calculated as a weighted average of the values computed on the individual classes. The accuracy per class was computed according to the following Equation (2):

(2) $$Accuracy=\frac{TP+TN}{TP+FP+TN+FN}$$

where TP (true positive) and FP (false positive) provide the number of images correctly and incorrectly classified as positive, respectively. On the other hand, TN (true negative) and FN (false negative) indicate the number of images classified as negative, correctly and incorrectly, respectively.

The experiments were performed on a workstation with the following hardware specifications: an Intel(R) Core(TM) i9-8950HK @ 2.90 GHz CPU, 32 GB RAM, and an NVIDIA GTX1050 Ti GPU with 4 GB of memory. All implementations and experimental evaluations were also performed in MATLAB R2021b. The source code with the moment’s implementation is freely available and was partially written by the same authors and available at http://bugs.unica.it/cana/software (accessed on 10 March 2023, see the orthomoms package), and partly obtained from a previous research article [38] https://github.com/ShurenQi/MomentToolbox (accessed on 10 March 2023).

### 5.2. Results

Here, we present a detailed account of the various experiments’ results.

For a precise evaluation of the individual descriptors, we report numerical tables detailing the results of the single descriptor categories obtained with each classifier. The classifier’s performance is briefly discussed later, as it is outside the scope of this work. To better examine the achieved results, we also report the feature vector dimensions (size column). We do not report the execution time since only some of the source code for the moment computation is optimised, which would lead to timelines that could be misleading. A detailed analysis of the execution time for our moments’ implementations has been reported in our previous work [25].

**Emphysema-CT.** The Emphysema-CT data set appears to be the most complex, based on the results obtained and shown in Table 3. Although the k-NN classifier performed best with 11 out of 26 descriptors (all moments), the results were much more varied. None of these 11 features was the absolute best, as deep features were in the top four, with the AlexNet features extracted by the SVM classifier being the absolute best (78.9%).

On average, deep features were by far the best category with an accuracy of 72%, followed by moments (Cartesian with 68.4%, Jacobian with 68%, Bessel–Fourier with 66.9%, and harmonic with 67.7%), and the texture features were last with an average of 63.3%.

Despite the excellent average performance achieved by the deep features, the moments were found to perform particularly well. The accuracy of 75% obtained by the BT classifier with the FrRHFM features was only 3.9% lower than the absolute best obtained by the features extracted from AlexNet (78.9%) and classified with the SVM. Specifically, FrRHFM was composed of a total of 66 features, 4,030 less than those of AlexNet. The k-NN classifier trained with FrPCET moments, consisting of 121 features, achieved the same accuracy of 75%.

**Brain-Tumor-MRI.** Contrary to the results on the previous data set, the absolute best results on Brain-Tumor-MRI were obtained by the k-NN classifier with 20 out of the 26 descriptors used (see Table 4). Apart from some exceptions caused by moments (JFM better by 1.5% with the SVM, HARri better by 1.7% with the BT), three out of four features extracted from the CNN (VGG19, ResNet50, GoogLeNet) gave better results with the SVM. However, the difference was not significant, except for GoogLeNet, which outperformed the k-NN classifier by 3.3% with the SVM.

On average, Cartesian moments were the best category with an accuracy of 93.7%, followed by the remaining moments: in order, Bessel–Fourier (92%), harmonic (91.8%), and Jacobian (91.7%). Thus, on average, all moments performed significantly better than the deep (90.8%) and texture (90.3%) descriptors.

This data set further illustrated how moments could produce particularly high results with few features. To give an example, the absolute best result was achieved by FrPCET and EFM coupled with a k-NN classifier (both with 98.2% accuracy) with 121 features, and the second best was achieved by CHM with a k-NN classifier (97.9% accuracy) with just 21 features.

**OASIS-MRI.** The first noteworthy observation from the OASIS MRI experiments is that all results were between 75 and 80%, as shown in Table 5, with a few exceptions, all provided by the NB classifier. In fact, it provided performance above 75% only with the FrPCET moments and AlexNet, VGG19, and ResNet50 features. On average, the best classifier was again the k-NN classifier, which performed best with 18 out of 26 descriptors, with both texture features and 15 out of 20 moments.

The best categories were the harmonic moments and the deep features with a 76.9% accuracy. However, unlike the previous data set, the categories were similar on average, as the Cartesian and Bessel–Fourier moments reached an accuracy of 76.5%, the Jacobian 76.3%, and the texture features 75.7%.

In general, the moments produced excellent results on this data set as well. Particularly noteworthy is the result obtained by the 66 features of CHFM with a k-NN classifier, which was the best overall with an accuracy of 80%.

**COVIDx CT-2A.** Regarding the COVIDx CT-2A data set, all the classifiers achieved an accuracy greater than 90% with any descriptor, except for the naive Bayes classifier, which achieved exceptionally high results (greater than 90%) only with features obtained from CNNs. As can be seen from Table 6, the best-performing classifier was the SVM, since it was able to obtain the best result with 23 of the 26 descriptors used, as well as being the best on average with an accuracy of 98.1%, 0.2% higher than the average accuracy obtained by the BT.

Moreover, among the identified descriptor categories, the one that performed best in this data set was the deep one, with an average accuracy of 96% among the features extracted from the four CNNs, followed by the texture features with 92.6%, and the Cartesian moments with 92%. In this context, it is crucial to note that the average of all the descriptors (except the deep ones) was strongly influenced by the results obtained by the naive Bayes classifier.

Therefore, we think it is particularly relevant to highlight, using the example of CH2M, how, with only 21 features, we were able to obtain an accuracy of 99%, which is very close to the best absolute performance of 99.4% obtained in this data set from the features extracted from VGG19 and classified by an SVM.

### 5.3. Discussion

**Figure 3:**
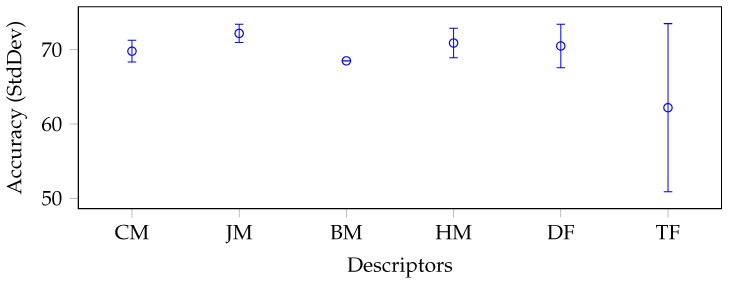
Average accuracy and standard deviation calculated on each descriptor category with the best classifier on average, for the Emphysema-CT data set.

**Figure 4:**
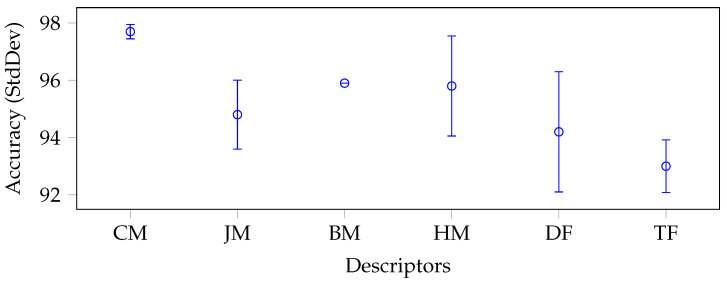
Average accuracy and standard deviation calculated on each descriptor category with the best classifier on average, for the Brain-Tumor-MRI data set.

**Figure 5:**
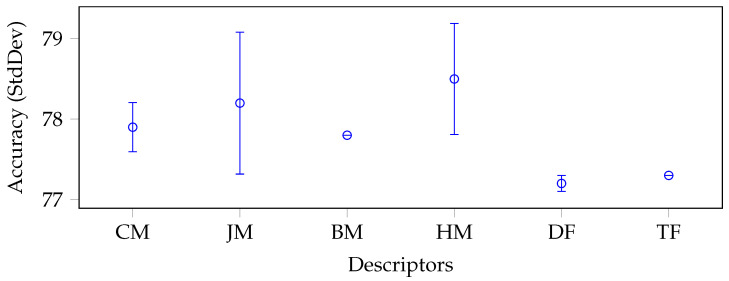
Average accuracy and standard deviation calculated on each descriptor category with the best classifier on average, for the Axial OASIS-MRI data set.

**Figure 6:**
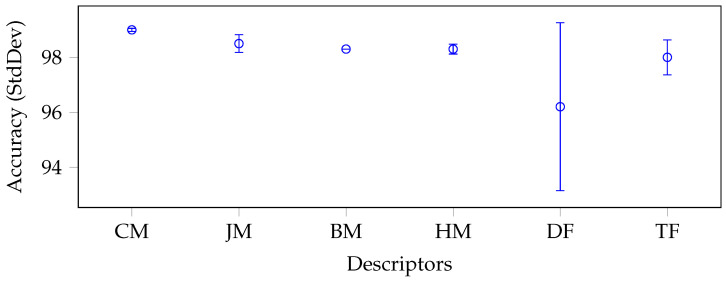
Average accuracy and standard deviation calculated on each descriptor category with the best classifier on average, for the COVIDx CT-2A data set.

To better understand and analyse the results regarding the performance of the identified moment categories, we report a whisker plot with confidence intervals for each data set in Figure 3, Figure 4, Figure 5 and Figure 6.

To produce such plots, we again grouped and reported on the x-axis the descriptors by categories: CM stands for Cartesian moments, JM for Jacobian moments, BM for Bessel–Fourier moments, HM for harmonic moments, DF for deep features, and TF for texture features. Each whisker along the y-axis represents the mean and standard deviation (minimum and maximum) accuracy value for a specific category of descriptors. The moments were generally very stable in each data set without considering the BM category, which consisted of only one descriptor. In fact, all categories of moments had a relatively low standard deviation, except for JM and HM in the Brain-Tumor-MRI (see Figure 4) and OASIS-MRI (see Figure 5) data sets.

If in the first case, the standard deviation was still lower than that produced by the deep features (1.20% and 1.75%, respectively, versus 2.1%), this trend was only partially confirmed in the second case. In this case, the deep features had a standard deviation of only 0.1% against 0.88% and 0.69%, respectively. In any case, these values were overall acceptable, considering the intrinsic complexity of the classes of the different data sets and, above all, the extremely small dimensions of their feature vectors compared to the deep ones. Moreover, with respect to the large COVIDx CT-2A data set (see Figure 6), where the classes were even closer, the deep features obtained unstable results (i.e., the standard deviation was 3%).

Last but not least, the Emphysema-CT data set was the one that showed a more pronounced difference between the deep features and the moments (see Figure 3). The moments performed better with the average best classifier (SVM) (FrPCET and FrRHFM obtained 75% as single descriptors). However, the absolute best result was obtained by the features extracted (with the k-NN classifier and BT, respectively) from VGG19 (which exceeded the absolute best obtained by the moments by 4.2%). In any case, such a difference could be accepted since the goal was to verify how much the moments were able to generalize their performance with respect to a specific task.

Considering the overall classification performance, recall that we optimized each individual classification algorithm since we wanted to study the absolute best performance of each descriptor. In this case, it can be observed that the naive Bayes classifier provided the worst results, being the classifier with the lowest average accuracy for all data sets. This trend was particularly emphasized for the COVIDx CT-2A data set, where the variance between the results obtained with moments was about 20% smaller than that obtained with deep features. However, even when observing the naive Bayes classifier in the other data sets, its performance when trained with moments tended to be between 6% and 9% lower than the best classifier. This behaviour is hypothetically due to the fact that naive Bayes assumes that the features considered are linearly independent. This aspect has been verified with deep features. In contrast, the features extracted with moments are highly correlated with each other, as can be seen in the work of Putzu et al. [89], where it is highlighted that feature selection applied to moments does not bring any benefit, unlike other categories of descriptors. This aspect provides additional robustness to their performance.

Finally, some useful guidelines emerged from the results of this work:

- The moments offered comparable performance to deep features, despite being represented by order of magnitude fewer features than deep features.
- The moments provided extremely robust results, with a reduced standard deviation per category in all the experiments performed.
- Cartesian and harmonic moments were behaved the best, both on average and in terms of the number of absolute best results they offered in the different experiments. They were also the most stable, with low standard deviations.
- There was no tendency for the moments to improve over the years; as mentioned in the previous point, the Cartesian moments performed best among the older ones. Moreover, the use of the fractional domain, one of the most recent innovations in the field, did not bring any significant advantage, as can be seen by comparing against the equivalent moments in the integer domain.
- The naive Bayes classifier was not suitable for the task under consideration, especially when trained by any category of moments, since the assumption that the features were linearly independent was too strong for this type of descriptors.
- Although moments allowed high accuracies for the SVM, k-NN and BT classifiers, the classifier that benefited the most from the moments was the k-NN classifier. It achieved significant absolute best results in all data sets except COVIDx CT-2A, where it had a marginal difference with the absolute top performer, the SVM.

## 6. Conclusions

This paper analysed the effectiveness of the best-known types of orthogonal moments in diagnostics when applied to diverse medical images. We first reviewed the state-of-the-art orthogonal moments, summarising their main categories and describing their main characteristics and differences. We then presented their main areas of application, with a particular emphasis on medical applications, which was the target of this work. We then evaluated orthogonal moments in different medical diagnostic tasks, highlighting the performance of orthogonal moments by comparing them with the best-known texture features and the latest CNNs features. This evaluation was carried out with five different classification techniques to guarantee that the results obtained were unrelated to the individual classifiers’ capabilities.

After analysing the experimental results presented in Section 5.3, we found that although CNNs showed impressive performance in all tasks, orthogonal moments proved to be a strong competitor, with comparable or even better results in some cases. It is worth noting that orthogonal moments require significantly fewer resources than CNNs, allowing them to be used in time-critical applications. In addition to being computationally expensive, deep learning approaches require large and diverse training sets and have limited robustness to geometric transformations, requiring data augmentation strategies to improve geometric invariance at the cost of time/space complexity. For these reasons, handcrafted features, particularly orthogonal moments, remain highly competitive in these domains. Orthogonal moments also outperform texture features, especially the well-known LBP descriptors.

Comparing the orthogonal moments among themselves, we observed that those defined in the Cartesian domain, although the least recent, performed the best. On the other hand, the more recent ones, of circular type and belonging to the fractional domain, did not seem to lead to further relevant benefits. It is not completely clear whether this trend was due to the different formulations or whether it was due to the order of moments used (see Section 4.2). From an empirical analysis, we observed that by increasing the order of the moments, the added features were irrelevant or with a low predictive power, but a thorough analysis of the moment order, even with fractional type orders, could be useful to confirm this trend.

Therefore, a challenging future investigation could be related to Cartesian moments on the fractional domain to see if they can bring more benefits to this task. Moreover, given their high performance, a further analysis could be relevant to verify the benefits of a combination of Cartesian moments. Another exciting direction of research could be related to the use of colour information, which is crucial for medical image analysis applications, but at the same time has been overlooked in the calculation of orthogonal moments. 

## Figures and Tables

**Table 1:** The year when the moments were first proposed.

Ref.	Descriptors	Year
[37]	Legendre moments	1980
[34]	Chebyshev moments of the first order	2001
[34]	Chebyshev moments of the second order	2001
[37]	Zernike moments	1980
[55]	Pseudo-Zernike moments	1988
[57]	Orthogonal Fourier–Mellin moments	1994
[58]	Chebyshev–Fourier moments	2002
[60]	Pseudo-Jacobi–Fourier moments	2004
[59]	Jacobi–Fourier moments	2007
[73]	Fractional-order Jacobi–Fourier moments	2021
[64]	Bessel–Fourier moments	2010
[61]	Radial harmonic Fourier moments	2003
[62]	Exponent Fourier moments	2014
[63]	Polar complex exponential transform	2010
[63]	Polar cosine transform	2010
[63]	Polar sine transform	2010
[71]	Fractional-order radial harmonic Fourier moments	2020
[68]	Fractional-order polar complex exponential transform	2014
[68]	Fractional-order polar cosine transform	2014
[68]	Fractional-order polar sine transform	2014

**Table 2:** The table provides information on the four different data sets used in this research. For each data set, the following details are given: number and size of the images, number of classes, and number of images per class.

Data Set	No. of Images	Size	Classes	No. of Images per Class
Emphysema-CT	168	61×61	3	59–50–59
Brain-Tumor-MRI	870	512×512	3	143–436–291
OASIS-MRI	436	176×208	4	336–70–28–2
COVIDx CT-2A	4942	512×512	3	3253–873–816

**Table 3:** Results obtained on the Emphysema-CT data set with all the analysed features, for which we report the categorisation (category column, CM—Cartesian moments, JM—Jacobian moments, BM—Bessel–Fourier moments, HM—harmonic moments, DF—deep features, and TF—texture features), the name (descriptor column), the feature size (size column), the accuracy obtained with all the tested classifiers (SVM, k-NN, DT, NB, BT columns), and the average accuracy value (AVG column). On the right (AVG cat.), we also report the average accuracy value of each category of features and on the bottom (AVG row), we report the average accuracy per classifier.

Category	Descriptor	Size	Classifiers					AVG	AVG Cat.
			SVM	k-NN	DT	NB	BT		
CM	LM	28	71.4	68.5	64.9	63.7	64.3	66.6	68.4
	CHM	21	69.6	69.6	70.2	63.7	69.0	68.4	
	CH2M	21	69.6	71.4	70.8	67.3	72.0	70.2	
JM	ZM	21	70.2	73.8	68.5	70.2	72.0	70.9	68.0
	PZM	36	71.4	70.8	66.7	64.9	67.3	68.2	
	OFMM	66	70.8	72.0	68.5	61.9	66.7	68.0	
	CHFM	66	64.3	72.0	72.0	61.3	63.7	66.7	
	PJFM	66	69.0	72.0	70.2	59.5	69.6	68.1	
	JFM	66	69.6	70.8	69.6	61.9	67.9	68.0	
	FrJFM	66	66.1	73.8	69.6	61.3	61.3	66.4	
BM	BFM	66	69.6	68.5	67.3	60.1	69.0	66.9	66.9
HM	RHFM	66	68.5	72.0	69.6	64.3	73.2	69.5	67.7
	EFM	121	67.3	70.2	67.3	63.7	67.9	67.3	
	PCET	121	65.5	67.9	70.8	58.9	69.0	66.4	
	PCT	66	65.6	72.0	69.0	60.7	66.7	66.8	
	PST	55	69.6	69.6	67.9	61.9	64.3	66.7	
	FrRHFM	66	70.2	70.8	73.2	66.7	75.0	71.2	
	FrPCET	121	66.7	75.0	70.8	58.9	69.0	68.1	
	FrPCT	66	64.9	70.2	67.9	61.9	63.1	65.6	
	FrPST	55	69.6	70.2	69.0	64.3	66.7	68.0	
DF	AlexNet	4096	78.9	73.8	63.1	72.6	76.8	73.0	72.0
	VGG19	4096	76.8	70.8	63.1	70.2	79.2	72.0	
	ResNet50	1000	78.6	70.8	64.3	75.0	77.4	73.2	
	GoogLeNet	1000	76.8	66.7	61.9	72.0	71.4	69.8	
TF	LBP18	36	53.0	54.2	56.5	54.2	56.5	54.9	63.3
	HARri	52	76.8	70.2	64.3	69.6	77.4	71.7	
	- AVG	-	69.6	70.3	67.6	64.3	69.1	-	

**Table 4:** Results obtained on the Brain-Tumor-MRI data set with all the analysed features, for which we report the categorisation (category column, CM—Cartesian moments, JM—Jacobian moments, BM—Bessel–Fourier moments, HM—harmonic moments, DF—deep features, and TF—texture features), the name (descriptor column), the feature size (size column), the accuracy obtained with all the tested classifiers (SVM, k-NN, DT, NB, BT columns) and the average accuracy value (AVG column). On the right (AVG cat.), we also report the average accuracy value of each category of features and on the bottom (AVG row), we report the average accuracy per classifier.

Category	Descriptor	Size	Classifiers					AVG	AVG Cat.
			SVM	k-NN	DT	NB	BT		
CM	LM	28	95.9	97.7	90.7	91.0	95.2	94.1	93.7
	CHM	21	94.6	97.9	89.7	91.0	94.7	93.6	
	CH2M	21	94.1	97.4	89.9	90.5	94.9	93.4	
JM	ZM	21	93.4	93.3	90.2	89.7	93.4	92.0	91.8
	PZM	36	94.1	94.9	90.5	89.9	94.8	92.8	
	OFMM	66	93.8	95.5	88.6	88.5	92.9	91.9	
	CHFM	66	93.7	96.2	87.1	87.7	93.7	91.7	
	PJFM	66	92.6	95.3	90.5	88.0	91.6	91.6	
	JFM	66	94.5	93.0	89.1	88.0	92.2	91.4	
	FrJFM	66	90.8	95.5	89.9	87.8	92.6	91.3	
BM	BFM	66	94.3	95.9	89.1	86.9	94.0	92.0	92.0
HM	RHFM	66	93.6	95.4	88.4	87.6	92.9	91.6	91.7
	EFM	121	94.9	98.2	87.1	86.7	92.6	91.9	
	PCET	121	96.7	97.7	87.4	86.7	92.0	92.1	
	PCT	66	92.2	94.4	87.4	88.3	91.4	90.7	
	PST	55	93.4	95.3	89.7	87.0	93.4	91.8	
	FrRHFM	66	94.1	95.2	89.2	88.3	92.0	91.8	
	FrPCET	121	95.9	98.2	89.4	87.0	94.6	93.0	
	FrPCT	66	94.0	94.5	88.4	87.6	91.5	91.2	
	FrPST	55	91.7	93.6	89.8	87.5	92.4	91.0	
DF	AlexNet	4096	94.6	94.7	88.6	83.9	93.1	91.0	90.8
	VGG19	4096	95.4	94.9	84.4	87.7	93.0	91.1	
	ResNet50	1000	95.7	95.9	88.5	87.4	93.8	92.3	
	GoogLeNet	1000	94.4	91.1	83.7	83.1	91.8	88.8	
TF	LBP18	36	92.3	93.6	89.4	77.1	90.3	88.5	90.3
	HARri	52	93.4	92.3	88.5	92.3	93.9	92.1	
	- AVG	-	94.0	95.3	88.7	87.6	93.0	-	-

**Table 5:** Results obtained on the OASIS-MRI data set with all the analysed features, for which we report the categorisation (category column, CM—Cartesian moments, JM—Jacobian moments, BM—Bessel–Fourier moments, HM—harmonic moments, DF—deep features, and TF—texture features), the name (descriptor column), the feature size (size column), the accuracy obtained with all the tested classifiers (SVM, k-NN, DT, NB, BT columns) and the average accuracy value (AVG column). On the right (AVG cat.), we also report the average accuracy value of each category of features and on the bottom (AVG row), we report the average accuracy per classifier.

Category	Descriptor	Size	Classifiers					AVG	AVG Cat.
			SVM	k-NN	DT	NB	BT		
CM	LM	28	77.8	78.2	77.1	71.8	77.5	76.5	76.5
	CHM	21	78.0	77.6	77.1	72.9	77.1	76.5	
	CH2M	21	77.5	78.0	77.1	72.5	77.1	76.4	
JM	ZM	21	77.3	78.2	77.1	70.6	77.3	76.1	76.3
	PZM	36	77.1	78.4	77.1	72.0	78.2	76.6	
	OFMM	66	77.1	78.2	77.1	72.5	77.1	76.4	
	CHFM	66	77.1	80.0	77.1	71.3	77.1	76.5	
	PJFM	66	77.1	77.8	77.1	70.6	75.7	75.7	
	JFM	66	77.1	78.0	78.0	71.8	77.8	76.5	
	FrJFM	66	78.2	77.1	77.3	72.5	77.1	76.4	
BM	BFM	66	77.1	77.8	77.1	74.1	76.6	76.5	76.5
HM	RHFM	66	77.1	78.4	78.4	72.0	76.6	76.5	76.9
	EFM	121	77.1	78.0	77.1	73.9	76.6	76.5	
	PCET	121	77.3	78.4	77.1	74.8	78.0	77.1	
	PCT	66	77.1	78.9	77.1	72.7	77.1	76.6	
	PST	55	77.1	79.4	79.6	72.7	79.1	77.6	
	FrRHFM	66	77.1	78.9	77.1	72.0	78.4	76.7	
	FrPCET	121	77.5	77.1	77.8	75.5	77.1	77.0	
	FrPCT	66	77.1	79.1	77.1	72.5	77.1	76.9	
	FrPST	55	77.3	78.2	79.1	72.2	77.1	76.8	
DF	AlexNet	4096	77.1	77.1	76.8	77.3	77.1	77.1	76.9
	VGG19	4096	78.7	77.1	75.7	77.5	78.9	77.6	
	ResNet50	1000	77.1	77.3	75.7	75.5	77.1	76.5	
	GoogLeNet	1000	78.4	77.1	77.1	72.9	76.4	76.4	
TF	LBP18	36	77.1	77.3	77.1	72.0	75.5	75.8	75.7
	HARri	52	77.1	77.3	77.1	69.7	77.1	75.7	
	- AVG	-	77.4	78.0	77.3	72.9	77.2	-	-

**Table 6:** Results obtained on the COVIDx CT-2A data set with all the analysed features, for which we report the categorisation (category column, CM—Cartesian moments, JM—Jacobian moments, BM—Bessel–Fourier moments, HM—harmonic moments, DF—deep features, and TF—texture features), the name (descriptor column), the feature size (size column), the accuracy obtained with all the tested classifiers (SVM, k-NN, DT, NB, BT columns) and the average accuracy value (AVG column). On the right (AVG cat.), we also report the average accuracy value of each category of features and on the bottom (AVG row), we report the average accuracy per classifier.

Category	Descriptor	Size	Classifiers					AVG	AVG Cat.
			SVM	k-NN	DT	NB	BT		
CM	LM	28	99.0	97.2	94.3	76.2	98.8	93.1	92.0
	CHM	21	99.0	96.9	93.9	72.0	98.7	92.1	
	CH2M	21	99.1	95.3	91.3	71.5	97.5	90.9	
JM	ZM	21	98.9	95.4	92.2	72.2	97.7	91.3	91.7
	PZM	36	99.0	96.7	93.4	75.8	98.4	92.7	
	OFMM	66	98.4	97.0	93.0	73.5	98.2	92.0	
	CHFM	66	98.3	97.0	92.8	72.5	98.2	91.8	
	PJFM	66	98.3	97.1	92.2	71.8	98.3	91.5	
	JFM	66	98.3	96.9	92.4	71.8	98.1	91.5	
	FrJFM	66	98.2	97.1	92.2	71.8	98.1	91.5	
BM	BFM	66	98.3	97.0	92.3	73.2	98.1	91.8	91.8
HM	RHFM	66	98.3	97.1	92.6	71.4	98.1	91.5	91.6
	EFM	121	98.3	97.6	91.5	70.3	98.2	91.2	
	PCET	121	98.0	97.7	92.5	72.1	98.4	91.7	
	PCT	66	98.4	97.2	93.1	72.2	98.5	91.9	
	PST	55	98.5	97.0	92.2	71.9	98.4	91.6	
	FrRHFM	66	98.3	97.1	92.6	71.4	98.2	91.5	
	FrPCET	121	98.1	97.6	92.2	72.5	98.3	91.7	
	FrPCT	66	98.5	97.3	93.0	72.3	98.4	91.9	
	FrPST	55	98.5	97.0	92.2	71.8	98.3	91.6	
DF	AlexNet	4096	97.5	97.5	97.4	97.4	97.5	97.5	96.0
	VGG19	4096	99.4	99.4	99.4	99.1	99.4	99.3	
	ResNet50	1000	95.8	95.8	95.6	95.0	96.5	95.7	
	GoogLeNet	1000	92.2	91.4	91.5	90.9	90.6	91.3	
TF	LBP18	36	98.4	98.0	95.0	74.1	97.9	92.7	92.4
	HARri	52	97.5	96.5	94.7	73.9	97.5	92.0	
	- AVG	-	98.1	96.8	93.3	76.1	97.9	-	-

## Data Availability

All the features extracted and the data produced in this study are available at the following url: https://github.com/andrealoddo/moments-for-medical-diagnosis GitHub repository (accessed on 10 March 2023).

## Abbreviations

The following abbreviations are used in this manuscript:

CAD	Computer-aided diagnostic
CNN	Convolutional neural network
VGGNet	Very deep convolutional networks
LBP	Local binary pattern
CT	Computed tomography
MR	Magnetic resonance
LM	Continuous Legendre moments
CHM	Discrete Chebyshev moments
CH2M	Discrete Chebyshev moments of the second order
ZM	Zernike moments
PZM	Pseudo-Zernike moments
OFMM	Orthogonal Fourier–Mellin moments
CHFM	Chebyshev–Fourier moments
PJFM	Pseudo-Jacobi–Fourier moments
JFM	Jacobi–Fourier moments
FrJFM	Fractional-order Jacobi–Fourier moments
BFM	Bessel–Fourier moments
RHFM	Radial harmonic Fourier moments
EFM	Exponent Fourier moments
PCET	Polar complex exponential transform
PCT	Polar cosine transform
PST	Polar sine transform
FrRHFM	Fractional-order radial harmonic Fourier moments
FrPCET	Fractional-order polar complex exponential transform
FrPCT	Fractional-order polar cosine transform
FrPST	Fractional-order polar sine transform
GLCM	Grey-level co-occurrence matrix
SVM	Support vector machine
k-NN	k-nearest neighbour
DT	Decision trees
NB	Naive Bayes
BT	Bagged trees
TP	True positive
FP	False positive
TN	True negative
FN	False negative
